# Impact of CD14^++^CD16^+^ monocytes on plaque vulnerability in diabetic and non-diabetic patients with asymptomatic coronary artery disease: a cross-sectional study

**DOI:** 10.1186/s12933-017-0577-8

**Published:** 2017-08-08

**Authors:** Naofumi Yoshida, Hiroyuki Yamamoto, Toshiro Shinke, Hiromasa Otake, Masaru Kuroda, Daisuke Terashita, Hachidai Takahashi, Kazuhiko Sakaguchi, Yushi Hirota, Takuo Emoto, Hilman Zulkifli Amin, Taiji Mizoguchi, Tomohiro Hayashi, Naoto Sasaki, Tomoya Yamashita, Wataru Ogawa, Ken-ichi Hirata

**Affiliations:** 1 Division of Cardiovascular Medicine, Department of Internal Medicine, Kobe University Graduate School of Medicine, 7-5-1 Kusunoki-cho, Chuo-ku, Kobe, Hyogo 6500017 Japan; 2 Division of Diabetes and Endocrinology, Department of Internal Medicine, Kobe University Graduate School of Medicine, 7-5-1 Kusunoki-cho, Chuo-ku, Kobe, Hyogo 6500017 Japan

**Keywords:** CD14^++^CD16^+^ monocytes, Coronary plaque vulnerability, Thin-cap fibroatheroma, Glucose fluctuations

## Abstract

**Background:**

Previously, we have reported that daily glucose fluctuations could affect coronary plaque vulnerability, but the underlying mechanisms remained unclear. This study sought to investigate the impact of CD14^++^CD16^+^ monocytes on plaque vulnerability, as assessed by virtual histology intravascular ultrasound (VH-IVUS), as well as their relationship to fluctuating glucose levels in patients with asymptomatic coronary artery disease (CAD).

**Methods:**

Fifty-one patients with asymptomatic CAD, who were undergoing lipid-lowering therapy and underwent VH-IVUS evaluation for angiographically mild to moderate lesions, were enrolled in the study. Standard VH-IVUS parameters, including the percentage volume of the necrotic core (%NC) within the plaque and the presence of a virtual histology thin-cap fibroatheroma (VH-TCFA), were then evaluated. Additionally, monocyte subsets were assessed by flow cytometry, and daily glucose fluctuations were analyzed by measuring the mean amplitude of glycemic excursion (MAGE).

**Results:**

Among 82 plaques from 22 diabetes mellitus (DM) patients and 29 non-DM patients, 15 VH-TCFAs were identified. CD14^++^CD16^+^ monocyte counts significantly correlated with both  %NC and the presence of VH-TCFA (%NC: r = 0.339, p = 0.002; VH-TCFA: p = 0.003). Multivariate logistic regression analysis revealed that CD14^++^CD16^+^ monocyte counts were independently associated with VH-TCFA (odds ratio = 1.029, p = 0.004). Furthermore, CD14^++^CD16^+^ monocyte counts were significantly correlated with the MAGE score in the non-DM patients (r = 0.544, p = 0.005).

**Conclusions:**

CD14^++^CD16^+^ monocyte levels are associated with coronary plaque vulnerability and can serve as a biomarker for VH-TCFA in patients with CAD undergoing lipid-lowering therapy. In patients without DM, glucose fluctuations may alter the balance of monocyte subsets.

*Trial registration* UMIN Registry number: UMIN000021228

**Electronic supplementary material:**

The online version of this article (doi:10.1186/s12933-017-0577-8) contains supplementary material, which is available to authorized users.

## Background

Recent studies have suggested that specific subsets of monocytes could play a critical role in the formation of atherosclerotic plaques [1]. Human monocytes can be divided into three subsets based on the CD14/CD16 expression: classical CD14^++^CD16^−^ monocytes, intermediate CD14^++^CD16^+^ monocytes, and non-classical CD14^+^CD16^+^ monocytes [2]. Classical CD14^++^CD16^−^ monocytes are phenotypically similar to Ly6C^high^/Gr-1^+^ mouse monocytes, with both groups expressing high levels of C–C chemokine receptor type 2 (CCR2), CD62L, and CD64 and low levels of CX3C chemokine receptor 1 (CX3CR1) [3]. Conversely, non-classical CD14^+^CD16^+^ monocytes express low levels of CCR2 and high levels of CX3CR1 and resemble Ly6C^low^/Gr-1^−^ mouse monocytes. Intermediate CD14^++^CD16^+^ monocytes selectively express C–C chemokine receptor type 5 (CCR5) [4], secret more inflammatory cytokines such as tumor necrosis factor-α and interleukin-1β [3], and have been shown to drive atherosclerosis [5]. Additionally, a large cohort study has reported that CD14^++^CD16^+^ monocytes independently predicted future cardiovascular events in subjects referred for elective coronary angiography (CAG) [1].

Previously, we have reported that daily glucose fluctuations could affect the coronary plaque vulnerability in coronary artery disease (CAD) patients undergoing lipid-lowering therapy [6, 7], but the mechanisms underlying this effect remain unclear. Certain clinical studies indicate a strong relation between glucose fluctuation and systemic inflammation, especially through the activation of toll-like receptors (TLR) [8–10]. Hence, we hypothesized that such fluctuations alter the prevalence of monocyte subsets, ultimately promoting coronary plaque vulnerability. In the present study, we investigated the impact of CD14^++^CD16^+^ monocytes on coronary plaque vulnerability and daily glucose fluctuations in patients with asymptomatic CAD, who were undergoing lipid-lowering therapy.

## Methods

### Patients

**Fig. 1:**
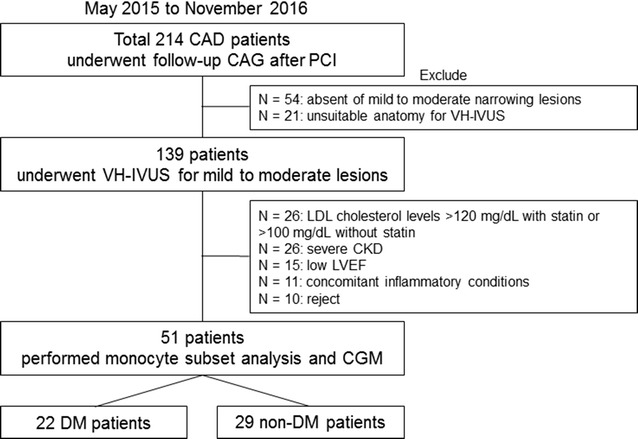
Study population. Fifty-one patients were enrolled in the study. *CAD* coronary artery disease, *CAG* coronary angiography, *CGM* continuous glucose monitoring, *CKD* chronic kidney disease, *DM* diabetes mellitus, *LDL* low-density lipoprotein, *LVEF* left ventricular ejection fractions, *PCI*, percutaneous coronary intervention, *VH*-*IVUS* virtual histology intravascular ultrasound, *VH*-*TCFA* virtual histology thin-cap fibroatheroma

In this study, we enrolled 214 consecutive patients who underwent follow-up CAG after a percutaneous coronary intervention, which is routinely carried out in Japan, between May 2015 and November 2016 (Fig. 1). Among these, 139 patients agreed to a virtual histology intravascular ultrasound (VH-IVUS) angiographic evaluation for angiographically mild to moderate lesions. Of the enrolled patients, those undergoing statin treatment with low-density lipoprotein (LDL) cholesterol levels below 120 mg/dL, and those undergoing other treatments for dyslipidemia with LDL cholesterol levels below 100 mg/dL, were deemed eligible for inclusion. However, patients with reduced left ventricular ejection fractions (<45%), renal disease (defined as serum creatinine >2.0 mg/dL), malignancies, or concomitant inflammatory conditions, including active infection, inflammatory arthritis, or connective tissue disease, were excluded from the study. Ultimately, a total of 51 patients underwent VH-IVUS for angiographically mild to moderate coronary lesions, monocyte subset analysis, and continuous glucose monitoring (CGM) as part of this study.

The patients were divided into two experimental groups according to the presence or absence of type 2 diabetes mellitus (DM). DM was defined by a clinical history, hemoglobin A1c levels ≥6.5%, and either a fasting plasma glucose level of ≥126 mg/dL or a plasma glucose level of ≥200 mg/dL 2 h after an oral glucose tolerance test [11]. This study was approved by the Ethics Committee of Kobe University and was carried out according to the guidelines set out in the Declaration of Helsinki. All subjects provided written informed consent before participating in this study.

### VH-IVUS examination and analysis

**Fig. 2:**
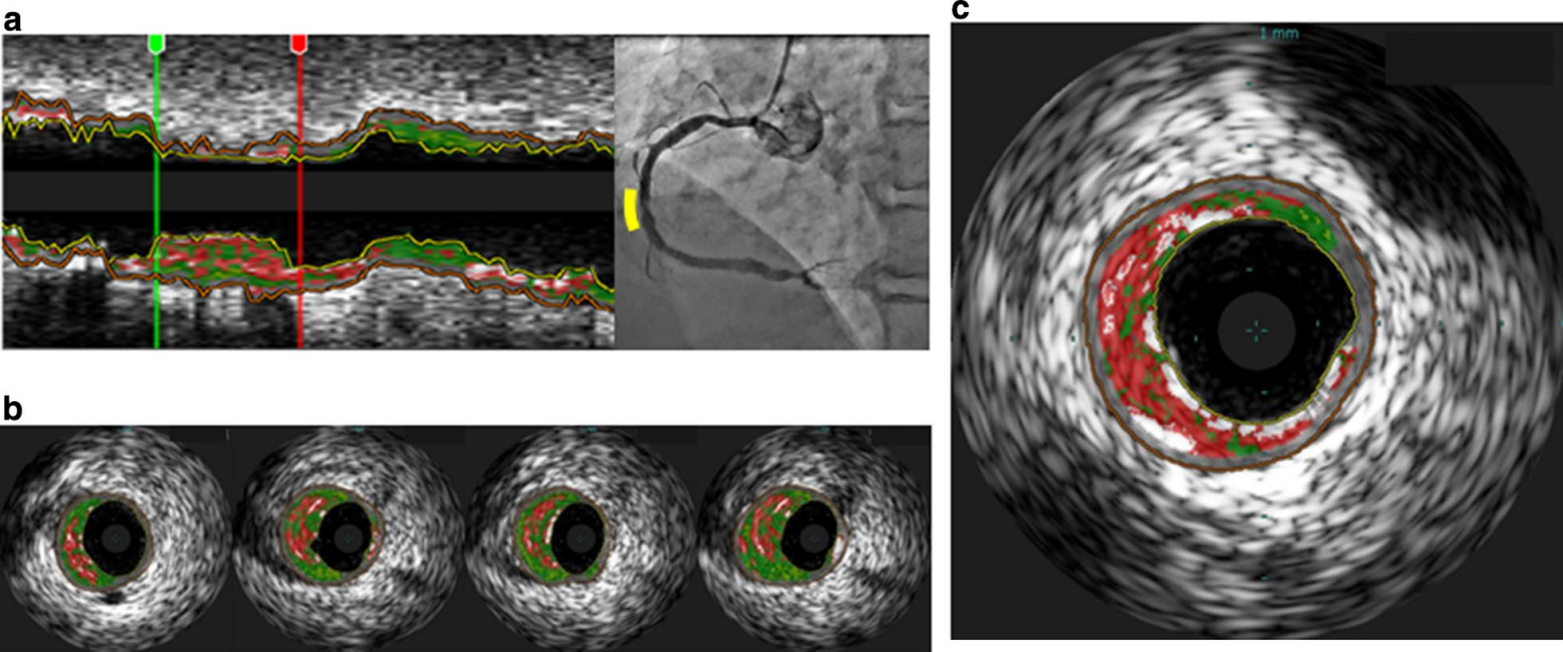
Representative longitudinal (**a**) and cross-sectional (**b**) VH-IVUS images for a lesion, assessed as part of the study. **c** A representative thin-cap fibroatheroma image. *VH*-*IVUS* virtual histology intravascular ultrasound

The VH-IVUS procedure was performed after intracoronary administration of 300 μg of nitroglycerin. First, a 20-MHz 2.9 Fr IVUS catheter (Eagle-Eye™; Volcano Therapeutics, Inc., Rancho Cordova, CA, USA) was introduced into the distal coronary artery. Then, using a motorized pullback device, the IVUS transducer was withdrawn at a rate of 0.5 mm/s until the coronary ostium was observed [12]. Lesions with angiographically mild to moderate stenosis, defined as a vessel diameter between 30 and 70% of the normal diameter, with a 30–70% plaque burden in the minimum lumen area of <4 mm^2^, as defined by IVUS, were analyzed as described previously [6] (Fig. 2a). Manual detection of contours in the lumen and at the media–adventitia interface was performed by blinded independent observers. The whole lesion volume was measured, and then the lumen, vessel, and plaque volumes were calculated using the Simpson’s method [6]. As reported previously, the VH-IVUS system automatically classified the plaque into four major components: fibrous (labeled green), fibrofatty (labeled greenish-yellow), necrotic core (NC; labeled red), and dense calcium (labeled white) regions [13] (Fig. 2b). The volume of each plaque component was then expressed as a percentage of the total plaque volume. The virtual histology thin-cap fibroatheroma (VH-TCFA; Fig. 2c) was defined as a lesion meeting the following criteria: (1) NC-rich (NC >10%), with no evidence of an overlying fibrous component and (2) a plaque volume >40% in at least three consecutive frames of VH-IVUS analysis [14]. These results were reviewed by the physician blinded to clinical data (HY).

### Blood sampling and flow cytometry analysis

**Fig. 3:**
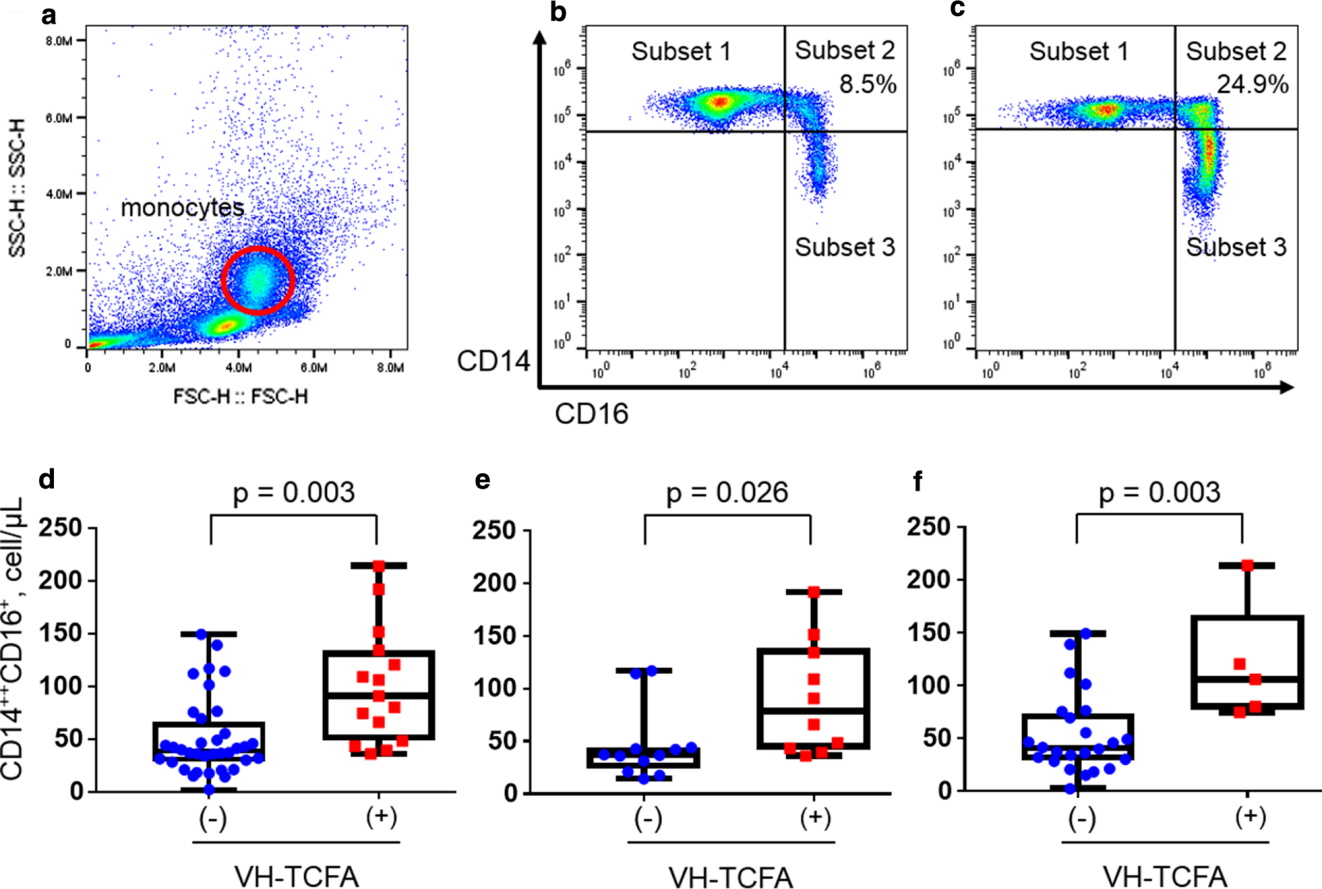
Flow cytometric analysis and the relationship between CD14^++^CD16^+^ monocyte levels and VH-TCFA prevalence. **a** First, FSC and SSC of the cells were measured, and the mononuclear cell population was gated (*red circle*). Next, the expression of CD14 and CD16 in the selected monocytes was assessed: **b** a representative plot from a patient with a low CD14^++^CD16^+^ rate; **c** a representative plot from a patient with a high CD14^++^CD16^+^ rate. Boxplots show CD14^++^CD16^+^ monocyte counts in all patients (**d**), DM patients (**e**), and non-DM patients (**f**) in the presence or absence of VH-TCFA. Error bars represent the minimum to maximum values. Subset 1: CD14^++^CD16^−^ monocytes. Subset 2: CD14^++^CD16^+^ monocytes. Subset 3: CD14^+^CD16^+^ monocytes. *DM* diabetes mellitus, *FSC* forward scatter, *SSC* side scatter, *VH*-*TCFA* virtual histology thin-cap fibroatheroma

Blood samples were collected in ethylenediaminetetraacetic acid-coated tubes after an overnight fast, and then peripheral blood mononuclear cells were isolated by Ficoll gradient centrifugation [15]. Isolated cells were stained with fluorescein isothiocyanate-labeled anti-CD14 (clone M5/E2; BD Biosciences), Alexa Fluor^®^ 647-labeled anti-CD16 (clone 3G8; BD Biosciences) antibodies, and isotype-matched control antibodies in phosphate-buffered saline containing 2% fetal calf serum according to standard protocols. Next, fluorescence-activated cell sorter analysis was performed using an Attune^®^ acoustic focusing cytometer (Life Technologies, Carlsbad, CA, USA) and the FlowJo software version 10.0.6 (Tree Star). We analyzed the flow cytometric data blinded to clinical data, and gated the monocyte subsets according to the staining with the isotype control. Representative flow cytometry data are shown in Fig. 3a–c.

### CGM system and analysis of daily glucose fluctuations

CGM was performed for 3 consecutive days. The data obtained on days 2 and 3 were used to establish daily blood glucose profiles since the insertion or removal of the sensor could lead to unreliable readings. For all patients, the CareLink iPro CGM analysis software (Medtronic, Northridge, CA, USA) was used to calculate the median values for the following variables on days 2 and 3: average glucose levels over 24 h, the time spent in a hyperglycemic or hypoglycemic state, and the mean amplitude of glycemic excursions (MAGE). MAGE, as described by Service et al. [16], represents fluctuations in the glucose level over a 24-h period and is calculated using variations in the glucose level measured continuously by CGM over a 2-day period. The time spent in either a hyperglycemic or hypoglycemic state was defined as the length of time during which blood glucose levels were >140 and <70 mg/dL, respectively. During hospitalization, all patients received nutritionally balanced meals (25–28 kcal/kg of the ideal body weight, 60% carbohydrate, 15–20% protein, and 20–25% fat).

### Statistical analysis

All data are presented as the mean ± standard deviation or as proportions. The averages of continuous variables were compared using either a two-tailed unpaired *t* test or a Mann–Whitney test, and either Chi square tests or Fisher’s exact test were used to compare the proportions of categorical variables between groups. Simple linear correlations between pairs of parameters were calculated using the least-squares method and by determining the Pearson’s correlation coefficient. Baseline variables with p < 0.2 in univariate logistic regression analyses were included in multivariate logistic regression analysis. Analyses were performed using commercially available software (SPSS version 22, IBM Corp., Armonk, NY, USA). Values of p < 0.05 were considered statistically significant.

## Results

### Baseline patient characteristics

The baseline characteristics of the 51 patients included in the study, including medications taken and laboratory data, are listed in Table 1. Of these 51 patients, 22 had DM and underwent medical treatment before admission. The duration of DM in these patients was 10.0 ± 2.1 years. All DM patients took antidiabetic medications (dipeptidyl peptidase-4 inhibitors, metformin, sulfonylurea, alpha-glucosidase inhibitors, pioglitazone, and glinide), whereas none of the non-DM patients took these medications. Four of the non-DM patients were excluded from blood glucose analysis because of a poor quality of the CGM data, and thus, 22 DM and 25 non-DM patients were included in the final CGM data analysis. The plasma LDL cholesterol levels were comparable between the DM and non-DM groups. Overall, the only significant differences observed between the DM and non-DM groups were related to their blood glucose profiles. The fasting blood sugar level, glycoalbumin level, glycated hemoglobin (HbA1c) level, and 1,5-anhydroglucitol level were all significantly higher in the DM patients, while the homeostasis model assessment of β-cell function index was significantly lower in the DM patients.

**Table 1:** Patient characteristics

	Total N = 51	DM N = 22	Non-DM N = 29	p value DM vs. non-DM
Age, years	70.2 ± 9.1	70.2 ± 9.2	70.2 ± 9.1	0.98
Male	38 (75)	17 (77)	21 (72)	0.69
BMI, kg/m^2^	23.7 ± 3.1	24.2 ± 2.6	23.3 ± 3.4	0.34
Systolic BP, mmHg	123.0 ± 12.5	122.6 ± 13.6	123.3 ± 11.7	0.86
Diastolic BP, mmHg	67.1 ± 9.4	65.3 ± 10.5	68.5 ± 8.4	0.24
eGFR, mL/min/1.73 m^2^	65.6 ± 17.8	64.0 ± 22.8	66.9 ± 13.1	0.57
CRP, mg/dL	0.10 ± 0.14	0.12 ± 0.16	0.08 ± 0.13	0.34
Total cholesterol, mg/dL	153.3 ± 24.9	150.1 ± 27.1	155.7 ± 23.4	0.43
LDL cholesterol, mg/dL	87.2 ± 20.1	84.7 ± 19.4	89.0 ± 20.8	0.45
HDL cholesterol, mg/dL	45.8 ± 12.5	44.9 ± 14.6	46.4 ± 10.9	0.68
Triglyceride, mg/dL	157.8 ± 84.7	161.2 ± 81.4	155.2 ± 88.6	0.80
Duration of DM, yrs	3.9 ± 1.1	10.0 ± 2.1	–	–
1,5-AG, μg/mL	16.0 ± 8.3	10.65 ± 6.51	20.23 ± 7.01	<0.01
FBS, mg/dL	104.9 ± 33.4	125.7 ± 41.2	89.1 ± 11.1	<0.01
Glycoalbumin, %	16.3 ± 3.3	18.7 ± 3.5	14.3 ± 1.1	<0.01
HbA1c, %	6.30 ± 0.76	6.95 ± 0.67	5.80 ± 0.31	<0.01
HOMA-R	2.01 ± 2.01	2.38 ± 2.29	1.74 ± 1.78	0.28
HOMA-β	100.4 ± 112.4	55.2 ± 38.1	133.1 ± 135.7	<0.01
LVEF, %	58.8 ± 8.5	61.0 ± 7.1	57.2 ± 9.3	0.12
Smoking, %				0.79
Current	7 (14)	3 (14)	4 (14)	
Past	23 (45)	11 (50)	12 (41)	
Past history				
Hypertension	40 (78)	20 (91)	20 (69)	0.06
Dyslipidemia	48 (94)	22 (100)	26 (90)	0.18
Medications on admission				
Aspirin	45 (88)	19 (86)	26 (90)	0.52
Thienopyridine	40 (78)	16 (73)	24 (83)	0.30
Statin	42 (82)	19 (86)	23 (79)	0.39
Beta-blocker	19 (37)	8 (36)	11 (38)	0.91
ACE-I/ARB	32 (63)	17 (77)	15 (52)	0.06
DPP4-I	16 (31)	16 (73)	0 (0)	<0.01
Metformin	7 (14)	7 (32)	0 (0)	<0.01
Sulfonylurea	1 (2)	1 (5)	0 (0)	0.43
α-GI	4 (8)	4 (18)	0 (0)	<0.01
Pioglitazone	1 (2)	1 (5)	0 (0)	0.43
Glinide	1 (2)	1 (5)	0 (0)	0.43

Data are represented as mean ± standard deviation, or as counts (%)

*1,5*-*AG* 1,5 anhydroglucitol, *α*-*GI* α-glucosidase inhibitor, *ACE*-*I* angiotensin-converting enzyme inhibitor, *ARB* angiotensin II receptor blocker, *BMI* body mass index, *BP* blood pressure, *CRP* C-reactive protein, *DM* diabetes mellitus, *DPP4*-*I* dipeptidyl peptidase-4 inhibitor, *FBS* fasting blood sugar, *eGFR* estimated glomerular filtration rate, *HbA1c* glycated hemoglobin, *HDL* high-density lipoprotein, *HOMA*-*β* homeostasis model assessment of β-cell function, *HOMA*-*R* homeostasis model assessment of insulin resistance, *LDL* low-density lipoprotein, *LVEF* left ventricular ejection fraction

### Plaque characteristics as determined by VH-IVUS

A total of 82 plaques were identified in the 51 patients, including 38 plaques in the 22 DM patients and 44 plaques in the 29 non-DM patients. The coronary plaque characteristics are summarized in Table 2. The plaque volume tended to be greater in the DM patients than in the non-DM patients, although the difference was not statistically significant (56.7 ± 7.6% vs. 54.1 ± 6.6%, respectively; p = 0.10). Similarly, the DM patients had a higher percentage of dense calcium than non-DM patients (12.6 ± 7.9% vs. 9.6 ± 6.6%, respectively; p = 0.06), as well as a higher absolute NC value (21.4 ± 18.2 mm^3^ vs. 14.6 ± 15.7 mm^3^, respectively; p = 0.07). However, the DM and non-DM patients exhibited similar percentages of fibrous components (53.5 ± 10.2% vs. 56.8 ± 10.4%, respectively; p = 0.15) and fibrofatty components (11.7 ± 5.0% vs. 13.2 ± 6.1%, respectively; p = 0.26). A higher incidence of VH-TCFA was observed in the DM patients than in the non-DM patients, although the difference was not statistically significant (26 vs. 11%, respectively; p = 0.08).

**Table 2:** Plaque characteristics, evaluated by VH-IVUS

	Total N = 82	DM N = 38	Non-DM N = 44	p value DM vs. non-DM
Plaque location				0.91
LAD	38 (46)	18 (47)	20 (45)	
LCx	16 (20)	6 (16)	10 (23)	
RCA	24 (29)	12 (32)	12 (27)	
LMT	4 (5)	2 (5)	2 (5)	
Plaque volume				
Absolute data, mm^3^	148.3 ± 114.8	148.3 ± 114.8	114.1 ± 108.4	0.17
Plaque burden, %	55.5 ± 7.1	56.7 ± 7.6	54.1 ± 6.6	0.10
Lesion length, mm	16.7 ± 13.2	17.9 ± 13.5	16.0 ± 12.2	0.51
Fibrous				
Absolute data, mm^3^	45.1 ± 46.5	50.6 ± 39.0	41.9 ± 50.6	0.39
Plaque burden, %	54.8 ± 10.6	53.5 ± 10.2	56.8 ± 10.4	0.15
Fibrofatty				
Absolute data, mm^3^	10.1 ± 10.4	11.8 ± 11.2	9.7 ± 10.6	0.41
Plaque burden, %	12.1 ± 5.6	11.7 ± 5.0	13.2 ± 6.1	0.26
Dense calcium				
Absolute data, mm^3^	10.0 ± 12.5	13.0 ± 15.3	6.6 ± 7.3	0.023
Plaque burden, %	11.5 ± 7.4	12.6 ± 7.9	9.6 ± 6.6	0.06
Necrotic core				
Absolute data, mm^3^	18.1 ± 17.8	21.4 ± 18.2	14.6 ± 15.7	0.07
Plaque burden, %	21.6 ± 6.8	22.2 ± 5.3	20.4 ± 7.9	0.25
Thin-cap fibroatheromas	15 (18)	10 (26)	5 (11)	0.08

Data are represented as mean ± standard deviation, or as counts (%)

*DM* diabetes mellitus, *LAD* left anterior descending coronary artery, *LCx* left circumflex artery, *LMT* left main trunk, *RCA* right coronary artery

### Relationship between coronary plaque properties and monocyte subsets

The cell counts and relative proportions of the different monocyte subsets are shown in Table 3. No significant differences were observed in either the cell counts or monocyte subset percentages between the DM and non-DM patients. Although not statistically significant, patients with higher CD14^++^CD16^+^ monocyte counts had higher plasma C-reactive protein concentrations (r = 0.182, p = 0.201).

**Table 3:** Monocyte subset counts and percentages

	Total N = 51	DM N = 22	Non-DM N = 29	p value DM vs. non-DM
Total monocyte, cell/μL	350.1 ± 180.3	355.9 ± 173.5	345.7 ± 188.1	0.84
CD14^++^CD16^−^, cell/μL	233.0 ± 126.8	232.3 ± 115.6	233.6 ± 136.8	0.97
CD14^++^CD16^+^, cell/μL	64.9 ± 47.4	65.5 ± 47.7	64.5 ± 48.0	0.94
CD14^+^CD16^++^, cell/μL	52.1 ± 34.7	58.1 ± 38.0	47.6 ± 32.0	0.29
CD14^++^CD16^−^, %	66.6 ± 12.4	66.5 ± 11.5	66.7 ± 13.2	0.95
CD14^++^CD16^+^, %	18.2 ± 9.0	17.6 ± 6.7	18.7 ± 10.4	0.68
CD14^+^CD16^++^, %	15.0 ± 6.1	15.8 ± 6.9	14.4 ± 5.4	0.44

Data are represented as mean ± standard deviation, or as counts (%)

*DM* diabetes mellitus

The CD14^++^CD16^+^ monocyte counts were significantly correlated with the percentage burden of fibrous components, NC, and dense calcium (fibrous components: r = −0.38, p < 0.01; NC: r = 0.34, p < 0.01; dense calcium: r = 0.29, p = 0.01) (Table 5). Furthermore, the CD14^++^CD16^+^ monocyte counts were significantly higher in the patients with VH-TCFA than in those without VH-TCFA, irrespective of the DM status (all patients: 100.3 ± 54.6 cells/μL vs. 50.8 ± 36.7 cells/μL, respectively, p = 0.003; DM patients: 91.0 ± 54.2 cells/μL vs. 46.2 ± 33.9 cells/μL, respectively, p = 0.026; non-DM patients: 119.0 ± 56.3 cells/μL vs. 53.1 ± 38.5 cells/μL, respectively, p = 0.003) (Fig. 3d–f).

### Relationship between glucose fluctuations and coronary plaque properties

The DM patients exhibited a significantly higher MAGE score than the non-DM patients (78.5 ± 14.4 mg/dL vs. 52.0 ± 13.2 mg/dL, respectively; p < 0.01) (Table 4). Indeed, the observed values for all other glycemic variables were significantly greater in the DM patients than in the non-DM patients, with the exception of the time spent in hypoglycemia, which tended to be longer in the non-DM patients than in the DM patients (Table 4). In non-DM patients, plasma C-reactive protein concentrations was higher in patients with hypoglycemia than in those without hypoglycemia (0.064 ± 0.013 mg/dL vs. 0.107 ± 0.051 mg/dL, respectively). The MAGE scores were significantly correlated with %NC (r = 0.339, p = 0.002) (Table 5). Moreover, the MAGE scores were significantly higher in the patients with VH-TCFA than in those without VH-TCFA (74.0 ± 16.9 mg/dL vs. 60.8 ± 20.0 mg/dL, respectively; p = 0.038) (Additional file 1: Figure S1). The coronary plaque properties were tested for simple linear correlations against laboratory variables (Table 5). Laboratory variables other than CD14^++^CD16^+^ monocyte counts and MAGE were not significantly correlated with coronary plaque properties (Table 5).

**Table 4:** Variables measured by the continuous glucose monitoring system

	Total N = 47	DM N = 22	Non-DM N = 25	p value DM vs. non-DM
MAGE, mg/dL	64.4 ± 19.1	78.5 ± 14.4	52.0 ± 13.2	<0.01
Mean blood glucose, mg/dL	130.2 ± 27.2	151.8 ± 23.5	111.1 ± 11.2	<0.01
Max blood glucose, mg/dL	222.9 ± 56.9	262.7 ± 43.2	187.8 ± 42.8	<0.01
Min blood glucose, mg/dL	80.2 ± 23.7	93.8 ± 23.2	68.2 ± 16.9	<0.01
Time in hyperglycemia, %	33.5 ± 31.1	59.5 ± 26.0	10.7 ± 10.0	<0.01
Time in hypoglycemia, %	3.5 ± 12.8	0.30 ± 0.9	6.3 ± 17.2	0.09

Data are represented as mean ± standard deviation. Time in hyperglycemia: length of time during which blood glucose levels >140 mg/dL. Time in hypoglycemia: length of time during which blood glucose levels <70 mg/dL

*DM* diabetes mellitus, *MAGE* mean amplitude of glycemic excursion

**Table 5:** Correlation of plaque properties and laboratory variables

	CD14^++^CD16^+^ monocytes	MAGE	CRP	LDL cholesterol	HDL cholesterol	HbA1c
Fibrous, %	−0.38 (0.001)	−0.31 (0.006)	−0.09 (0.45)	0.03 (0.78)	−0.10 (0.42)	−0.11 (0.37)
Fibrofatty, %	−0.08 (0.46)	−0.05 (0.028)	0.07 (0.55)	0.14 (0.23)	−0.13 (0.28)	−0.15 (0.20)
Necrotic core, %	0.34 (0.002)	0.34 (0.009)	−0.06 (0.61)	−0.14 (0.23)	0.03 (0.82)	0.13 (0.26)
Dense calcium, %	0.29 (0.009)	0.30 (0.003)	0.13 (0.26)	−0.02 (0.85)	0.21 (0.08)	0.14 (0.23)

Values represent r values (p values)

*CRP* C-reactive protein, *HbA1c* glycated hemoglobin, *HDL* high-density lipoprotein, *LDL* low-density lipoprotein, *MAG*E mean amplitude of glycemic excursion

### Relationship between glucose fluctuations and monocyte subsets

**Fig. 4:**
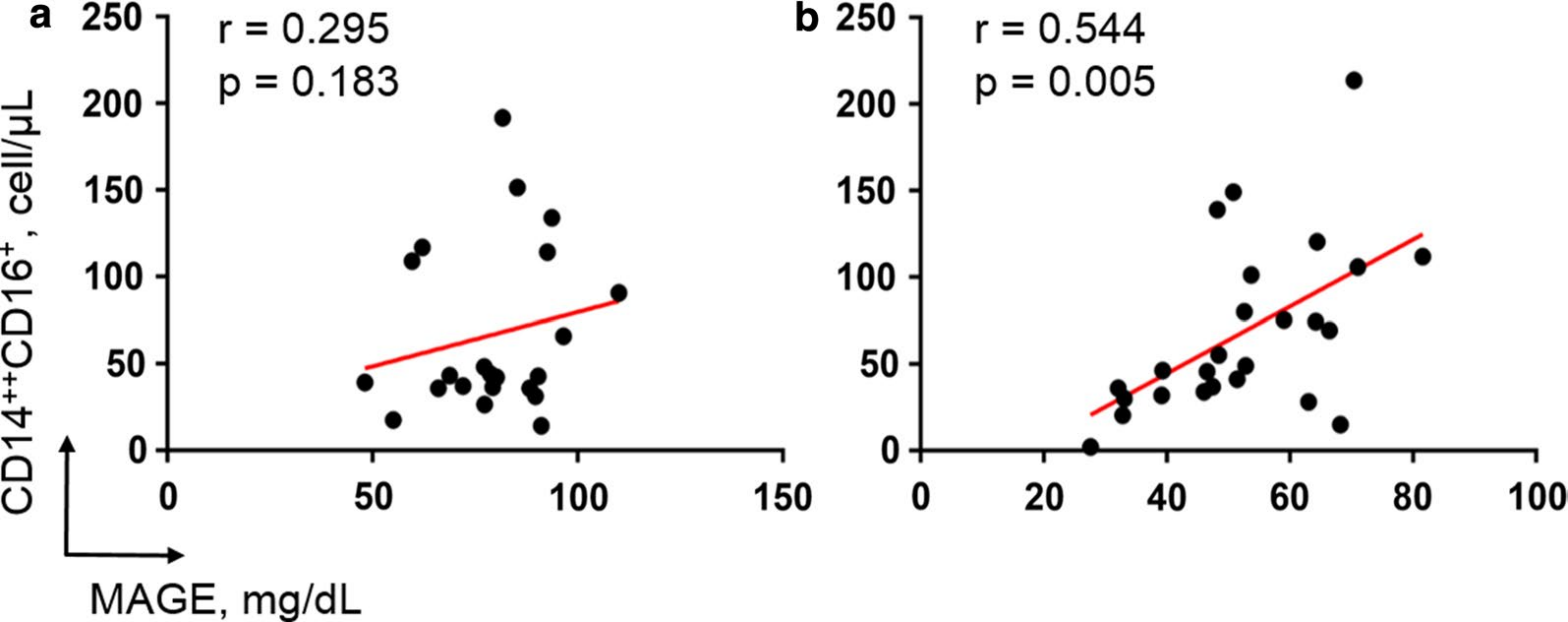
Impact of glucose fluctuation on CD14^++^CD16^+^ monocytes. Scatterplots show the relationship between daily glucose fluctuation (MAGE score; mg/dL) and CD14^++^CD16^+^ monocyte counts in DM patients (**a**) and non-DM patients (**b**). Pearson’s coefficients (r) and p values are shown above each plot. *DM* diabetes mellitus, *MAGE* mean amplitude of glycemic excursion

There was no significant correlation between the CD14^++^CD16^+^ monocyte counts and MAGE scores when either the total patient population or the DM patient population was considered (all patients: r = 0.280, p = 0.056; DM patients: r = 0.295, p = 0.183) (Fig. 4a). Interestingly, however, the CD14^++^CD16^+^ monocyte counts were significantly correlated with the MAGE scores in the non-DM patients (r = 0.544, p = 0.005) (Fig. 4b).

### Association between VH-TCFA and diagnostic variables

To identify independent risk factors for VH-TCFA, logistic regression analysis was applied. First, we conducted univariate logistic regression analyses to identify potential risk factors for VH-TCFA using the presence of DM, the MAGE score, time in hyperglycemia, and CD14^++^CD16^+^ monocyte counts. We then used any variables defined as p < 0.2 in these analyses to conduct a multivariate logistic regression analysis. We found that in all plaques, CD14^++^CD16^+^ monocyte counts were the independent predictor of VH-TCFA (odds ratio: 1.029; 95% confidence interval 1.009 to 1.049; p = 0.004) (Table 6).

**Table 6:** Univariate and multivariate logistic regression analyses as contributors to the VH-TCFA

	Univariate analysis			Multivariate analysis		
	OR	95% CI	p value	OR	95% CI	p value
Presence of DM	4.0	1.115–14.354	0.033	6.001	1.144–31.595	0.034
MAGE, mg/dL	1.047	1.007–1.088	0.021			
Time in hyperglycemia, %	1.008	0.998–1.028	0.441			
CD14^++^CD16^+^ monocytes, cell/μL	1.025	1.009–1.042	0.003	1.029	1.009–1.049	0.004

CI confidence interval, *DM* diabetes mellitus, *MAGE* mean amplitude of glycemic excursion, *OR* odds ratio

## Discussion

Recently, it has been suggested that specific monocyte subsets play a vital and complex role in the atherosclerotic plaque formation [1, 17]. A study in mice showed that Ly6C^high^ monocytes, the main precursors of M1 macrophages, adhered to the vascular endothelium, infiltrated the vessel wall, and contributed to plaque progression [18]. Conversely, Ly6C^low^ monocytes, the main precursors of M2 macrophages, were not found to directly contribute to plaque progression. However, results from animal experiments could not be reproduced with human monocytes [19], and thus, the role of monocytes in human atherogenesis remains unclear.

A cross-sectional study investigated the impact of CD14^++^CD16^+^ monocytes on coronary plaques in patients with acute myocardial infarction showed that CD14^++^CD16^+^ monocytes were significantly more prevalent in patients with coronary plaque rupture than in those without plaque rupture [20]. Similar to that observed in patients with CAD, CD14^++^CD16^+^ monocyte levels increased in patients with peripheral artery occlusive disease [21]. However, the impact of CD14^++^CD16^+^ monocytes on the features of the coronary plaque and it’s vulnerability, has not been previously evaluated. We, therefore, used VH-IVUS in asymptomatic patients undergoing lipid-lowering therapy to show, for the first time, that the CD14^++^CD16^+^ monocyte level correlates with plaque vulnerability. These monocytes could, therefore, be used as a surrogate marker of coronary plaque vulnerability and could potentially be specifically targeted in novel strategies for atherosclerosis treatment. Increased CD14^++^CD16^+^ monocyte levels were found to be an independent predictor of TCFA, which is itself considered to be a precursor of plaque rupture. Decreasing CD14^++^CD16^+^ monocyte levels would, therefore, be expected to therapeutically stabilize the vulnerable coronary plaques and reduce the probability of plaque rupture.

One potential mechanism by which CD14^++^CD16^+^ monocytes affect the plaque vulnerability is via the surface expression of CCR5, which has been shown to be a pro-atherogenic chemokine receptor in both animal and human studies [22, 23]. Moreover, CD14^++^CD16^+^ monocytes secrete high levels of tumor necrosis factor-α and interleukin-1β, which are involved in the pathogenesis and progression of atherosclerosis [3, 24]. Alternatively, myeloperoxidase expression may be involved. CD14^++^CD16^+^ monocytes have been shown to have high levels of intracellular myeloperoxidase expression [21, 24], which is associated with atherosclerotic plaque progression and rupture [25, 26]. Furthermore, high levels of myeloperoxidase promote endothelial cell death, activate matrix metalloproteinases, and are a predictor of plaque vulnerability [27, 28].

Recent clinical studies showed that besides continuous hyperglycemia, large glucose fluctuation is associated with the development of cardiovascular disease, increased risk of acute coronary events, heterogeneous neointimal growth after everolimus-eluting stent implantation, and cardiovascular autonomic neuropathy in patients with DM or impaired glucose tolerance [20, 29–32]. These results suggested that both reduction and stable maintenance of blood glucose level should be targets of diabetes management. However, the precise mechanism by which daily glucose fluctuation deteriorate coronary plaque vulnerability was not known. A previous in vitro study showed enhanced apoptosis of human umbilical vein endothelial cells, which were intermittently, rather than continuously, exposed to high glucose concentrations [33]. Moreover, an in vivo study showed that fluctuations in blood glucose levels caused oxidative stress and inflammation in rat endothelial cells [34]. Additionally, in rats, fluctuations of glucose levels enhanced the adhesion of monocytes to the endothelium via the activation and upregulation of adhesion molecules on endothelial cells [35]. These results have suggested that glucose fluctuations could promote plaque vulnerability by damaging endothelial cells. While the exact mechanisms by which fluctuating glucose levels exert deleterious effects on CD14^++^CD16^+^ monocytes are unclear, previous studies imply a relationship between glucose fluctuations and systemic inflammation involving monocytes [8–10, 36, 37]. For instance, an intervention against glucose fluctuation with acarbose treatment significantly decreased inflammatory cytokines levels followed by reduced serum lipopolysaccharides levels [10]. Considering TLR4, a receptor of lipopolysaccharides, is more expressed on CD14^++^CD16^+^ monocytes [38], the link between glucose fluctuation and CD14^++^CD16^+^ monocytes is probably existed. Also, a recent ex vitro study showed that hypoglycemia promotes the mobilization of specific leukocyte subsets from the marginal pool and induces proinflammatory functional changes in peripheral blood mononuclear cells [39]. Given that hypoglycemia, assessed by CGM, is associated with atherosclerosis in non-DM patients [40], hypoglycemia and associated glucose fluctuations could explain the significant correlation that was observed in the non-DM patients between the MAGE scores and CD14^++^CD16^+^ monocyte levels. Taken together, these data suggest that fluctuating glucose levels could potentially alter the prevalence of monocyte subsets, leading to plaque vulnerability.

Antidiabetic medications such as dipeptidyl peptidase-4 inhibitors and metformin have been shown to reduce inflammation and monocyte recruitment [41–43]. We observed a weaker correlation between MAGE scores and CD14^++^CD16^+^ monocyte levels in the DM patients, who took antidiabetic drugs, than in the non-DM patients, who did not take antidiabetic drugs. Indeed, a strong positive correlation between MAGE scores and CD14^++^CD16^+^ monocyte levels was seen in the non-DM patients. It is speculated that fluctuations in glucose levels could affect plaque vulnerability in patients without DM by increasing the prevalence of CD14^++^CD16^+^ monocytes, and thus interventions for controlling fluctuating glucose levels may improve plaque stability in patients with a high MAGE score and without DM.

It is well known that lipid metabolism is altered dramatically in DM patients compared with non-DM patients. It is possible that the reduced lipoprotein lipase function in DM patients leads to increases in remnant lipoprotein and small dense LDL levels, as well as to a decrease in high-density lipoprotein, which together promote the development of vulnerable plaques. While higher fluctuations in glucose levels were observed in the DM patients, the association between such glucose fluctuation and plaque vulnerability may be confounded by factors such as lipid disorders. Indeed, the IMPROVE-IT observational study has indicated that aggressive lipid-lowering therapy may be more effective than interventions to reduce glucose fluctuations in preventing future coronary events [44, 45].

## Limitations

The present study had several limitations, which should be considered when interpreting the results. First, this was a single-center study with a relatively small number of patients. Additional multi-center studies with a larger number of patients will be needed to confirm these observations. Second, the only monocyte cell-surface molecules that were considered were CD14 and CD16. Third, duration of DM and antidiabetic medications may affect the MAGE score and monocyte subsets in patients with DM. Finally, since this study was a cross-sectional study, we cannot definitively state whether increasing CD14^++^CD16^+^ monocyte levels are a cause or the result of coronary plaque vulnerability and blood glucose fluctuations throughout the day. A further study is needed to confirm the hypothesis that increased CD14^++^CD16^+^ monocyte levels promote coronary plaque vulnerability.

## Conclusions

CD14^++^CD16^+^ monocyte levels closely correlated with plaque vulnerability in asymptomatic CAD patients, even when the patients were undergoing lipid-lowering therapy. Daily glucose fluctuation may potentially alter the balance of monocyte subsets, especially in patients with presymptomatic DM.

## Additional file

**Additional file 1.** Relationship between MAGE and VH-TCFA prevalence. Boxplots show MAGE in all patients (a), DM patients (b), and non-DM patients (c) in the presence or absence of VH-TCFA. Error bars represent the minimum to maximum values. *DM* diabetes mellitus, *MAGE* mean amplitude of glycemic excursion, *VH*-*TCFA* virtual histology thin-cap fibroatheroma.

## Abbreviations

CAD: coronary artery disease
CCR2: C–C chemokine receptor type 2
CCR5: C–C chemokine receptor type 5
CGM: continuous glucose monitoring
CX3CR1: CX3C chemokine receptor 1
DM: diabetes mellitus
HbA1c: glycated hemoglobin
LDL: low-density lipoprotein
MAGE: mean amplitude of glycemic excursion
NC: necrotic core
VH-IVUS: virtual histology intravascular ultrasound
VH-TCFA: virtual histology thin-cap fibroatheroma

## References

[1] Rogacev KS, Cremers B, Zawada AM, Seiler S, Binder N, Ege P (2012). CD14^++^CD16^+^ monocytes independently predict cardiovascular events: a cohort study of 951 patients referred for elective coronary angiography. J Am Coll Cardiol.

[2] Ziegler-Heitbrock L, Ancuta P, Crowe S, Dalod M, Grau V, Hart DN (2010). Nomenclature of monocytes and dendritic cells in blood. Blood.

[3] Cros J, Cagnard N, Woollard K, Patey N, Zhang SY, Senechal B (2010). Human CD14^dim^ monocytes patrol and sense nucleic acids and viruses via TLR7 and TLR8 receptors. Immunity.

[4] Zawada AM, Rogacev KS, Rotter B, Winter P, Marell RR, Fliser D (2011). SuperSAGE evidence for CD14^++^CD16^+^ monocytes as a third monocyte subset. Blood.

[5] Rogacev KS, Seiler S, Zawada AM, Reichart B, Herath E, Roth D (2011). CD14^++^CD16^+^ monocytes and cardiovascular outcome in patients with chronic kidney disease. Eur Heart J.

[6] Kuroda M, Shinke T, Sakaguchi K, Otake H, Takaya T, Hirota Y (2015). Effect of daily glucose fluctuation on coronary plaque vulnerability in patients pre-treated with lipid-lowering therapy: a prospective observational study. JACC Cardiovasc Interv.

[7] Kuroda M, Shinke T, Sakaguchi K, Otake H, Takaya T, Hirota Y (2015). Association between daily glucose fluctuation and coronary plaque properties in patients receiving adequate lipid-lowering therapy assessed by continuous glucose monitoring and optical coherence tomography. Cardiovasc Diabetol.

[8] Monnier L, Mas E, Ginet C, Michel F, Villon L, Cristol JP (2006). Activation of oxidative stress by acute glucose fluctuations compared with sustained chronic hyperglycemia in patients with type 2 diabetes. JAMA.

[9] Zhang Q, Xiao X, Li M, Li W, Yu M, Zhang H (2013). Acarbose reduces blood glucose by activating miR-10a-5p and miR-664 in diabetic rats. PLoS ONE.

[10] Su B, Liu H, Li J, Sunli Y, Liu B, Liu D (2015). Acarbose treatment affects the serum levels of inflammatory cytokines and the gut content of bifidobacteria in Chinese patients with type 2 diabetes mellitus. J Diabetes.

[11] Rydén L, Grant PJ, Anker SD, Berne C, Cosentino F, Authors/Task Force Members (2013). ESC guidelines on diabetes, pre-diabetes, and cardiovascular diseases developed in collaboration with the EASD: the Task Force on diabetes, pre-diabetes, and cardiovascular diseases of the European Society of Cardiology (ESC) and developed in collaboration with the European Association for the Study of Diabetes (EASD). Eur Heart J.

[12] Sawada T, Shite J, Shinke T, Otake H, Tanino Y, Ogasawara D (2010). Low plasma adiponectin levels are associated with presence of thin-cap fibroatheroma in men with stable coronary artery disease. Int J Cardiol.

[13] Nair A, Kuban BD, Tuzcu EM, Schoenhagen P, Nissen SE, Vince DG (2002). Coronary plaque classification with intravascular ultrasound radiofrequency data analysis. Circulation.

[14] Rodriguez-Granillo GA, Garcia-Garcia HM, Mc Fadden EP, Valgimigli M, Aoki J, de Feyter P (2005). In vivo intravascular ultrasound-derived thin-cap fibroatheroma detection using ultrasound radiofrequency data analysis. J Am Coll Cardiol.

[15] Suzuki A, Fukuzawa K, Yamashita T, Yoshida A, Sasaki N, Emoto T (2017). Circulating intermediate CD14^++^CD16^+^ monocytes are increased in patients with atrial fibrillation and reflect the functional remodelling of the left atrium. Europace.

[16] Service FJ, Molnar GD, Rosevear JW, Ackerman E, Gatewood LC, Taylor WF (1970). Mean amplitude of glycemic excursions, a measure of diabetic instability. Diabetes.

[17] Woollard KJ, Geissmann F (2010). Monocytes in atherosclerosis: subsets and functions. Nat Rev Cardiol.

[18] Swirski FK, Libby P, Aikawa E, Alcaide P, Luscinskas FW, Weissleder R (2007). Ly-6Chi monocytes dominate hypercholesterolemia-associated monocytosis and give rise to macrophages in atheromata. J Clin Investig.

[19] Gordon S, Taylor PR (2005). Monocyte and macrophage heterogeneity. Nat Rev Immunol.

[20] Teraguchi I, Imanishi T, Ozaki Y, Tanimoto T, Orii M, Shiono Y (2014). Impact of glucose fluctuation and monocyte subsets on coronary plaque rupture. Nutr Metab Cardiovasc Dis.

[21] Wildgruber M, Aschenbrenner T, Wendorff H, Czubba M, Glinzer A, Haller B (2016). The “intermediate” CD14^++^CD16^+^ monocyte subset increases in severe peripheral artery disease in humans. Sci Rep.

[22] Weber C, Noels H (2011). Atherosclerosis: current pathogenesis and therapeutic options. Nat Med.

[23] Pai JK, Kraft P, Cannuscio CC, Manson JE, Rexrode KM, Albert CM (2006). Polymorphisms in the CC-chemokine receptor-2 (CCR2) and -5 (CCR5) genes and risk of coronary heart disease among US women. Atherosclerosis.

[24] Wildgruber M, Czubba M, Aschenbrenner T, Wendorff H, Hapfelmeier A, Glinzer A (2016). Increased intermediate CD14^++^CD16^++^ monocyte subset levels associate with restenosis after peripheral percutaneous transluminal angioplasty. Atherosclerosis.

[25] Kataoka Y, Shao M, Wolski K, Uno K, Puri R, Tuzcu EM (2014). Myeloperoxidase levels predict accelerated progression of coronary atherosclerosis in diabetic patients: insights from intravascular ultrasound. Atherosclerosis.

[26] Tavora FR, Ripple M, Li L, Burke AP (2009). Monocytes and neutrophils expressing myeloperoxidase occur in fibrous caps and thrombi in unstable coronary plaques. BMC Cardiovasc Disord.

[27] Hazen SL (2004). Myeloperoxidase and plaque vulnerability. Arterioscler Thromb Vasc Biol.

[28] Ferrante G, Nakano M, Prati F, Niccoli G, Mallus MT, Ramazzotti V (2010). High levels of systemic myeloperoxidase are associated with coronary plaque erosion in patients with acute coronary syndromes: a clinicopathological study. Circulation.

[29] Teraguchi I, Imanishi T, Ozaki Y, Tanimoto T, Ueyama M, Orii M (2014). Acute-phase glucose fluctuation is negatively correlated with myocardial salvage after acute myocardial infarction. Circ J.

[30] Jun JE, Jin S-M, Baek J, Oh S, Hur KY, Lee M-S (2015). The association between glycemic variability and diabetic cardiovascular autonomic neuropathy in patients with type 2 diabetes. Cardiovasc Diabetol.

[31] Okada K, Hibi K, Gohbara M, Kataoka S, Takano K, Akiyama E (2015). Association between blood glucose variability and coronary plaque instability in patients with acute coronary syndromes. Cardiovasc Diabetol.

[32] Kuroda M, Shinke T, Otake H, Sugiyama D, Takaya T, Takahashi H (2016). Effects of daily glucose fluctuations on the healing response to everolimus-eluting stent implantation as assessed using continuous glucose monitoring and optical coherence tomography. Cardiovasc Diabetol.

[33] Risso A, Mercuri F, Quagliaro L, Damante G, Ceriello A (2001). Intermittent high glucose enhances apoptosis in human umbilical vein endothelial cells in culture. Am J Physiol Endocrinol Metab.

[34] Wu N, Shen H, Liu H, Wang Y, Bai Y, Han P (2016). Acute blood glucose fluctuation enhances rat aorta endothelial cell apoptosis, oxidative stress and pro-inflammatory cytokine expression in vivo. Cardiovasc Diabetol.

[35] Azuma K, Kawamori R, Toyofuku Y, Kitahara Y, Sato F, Shimizu T (2006). Repetitive fluctuations in blood glucose enhance monocyte adhesion to the endothelium of rat thoracic aorta. Arterioscler Thromb Vasc Biol.

[36] Hartstra AV, Bouter KE, Bäckhed F, Nieuwdorp M (2015). Insights into the role of the microbiome in obesity and type 2 diabetes. Diabetes Care.

[37] Pedersen HK, Gudmundsdottir V, Nielsen HB, Hyotylainen T, Nielsen T, Jensen BAH (2016). Human gut microbes impact host serum metabolome and insulin sensitivity. Nature.

[38] Ozaki Y, Imanishi T, Hosokawa S, Nishiguchi T, Taruya A, Tanimoto T (2017). Association of toll-like receptor 4 on human monocyte subsets and vulnerability characteristics of coronary plaque as assessed by 64-slice multidetector computed tomography. Circ J.

[39] Ratter JM, Rooijackers HM, Tack CJ, Hijmans AG, Netea MG, de Galan BE (2017). Pro-inflammatory effects of hypoglycemia in humans with or without diabetes. Diabetes.

[40] Castaldo E, Sabato D, Lauro D, Sesti G, Marini MA (2011). Hypoglycemia assessed by continuous glucose monitoring is associated with preclinical atherosclerosis in individuals with impaired glucose tolerance. PLoS ONE.

[41] Hattori Y, Suzuki K, Hattori S, Kasai K (2006). Metformin inhibits cytokine-induced nuclear factor κB activation via AMP-activated protein kinase activation in vascular endothelial cells. Hypertension.

[42] Fadini GP, Boscaro E, Albiero M, Menegazzo L, Frison V, de Kreutzenberg S (2010). The oral dipeptidyl peptidase-4 inhibitor sitagliptin increases circulating endothelial progenitor cells in patients with type 2 diabetes: possible role of stromal-derived factor-1α. Diabetes Care.

[43] Zhong J, Maiseyeu A, Davis SN, Rajagopalan S (2015). DPP4 in cardiometabolic disease: recent insights from the laboratory and clinical trials of DPP4 inhibition. Circ Res.

[44] Cannon CP, Blazing MA, Giugliano RP, McCagg A, White JA, Theroux P (2015). Ezetimibe added to statin therapy after acute coronary syndromes. N Engl J Med.

[45] Feingold KR, Grunfeld C, Pang M, Doerrler W, Krauss RM (1992). LDL subclass phenotypes and triglyceride metabolism in non-insulin-dependent diabetes. Arterioscler Thromb.

