# Supplementary figures and images for: Impact of CD14++CD16+ monocytes on plaque vulnerability in diabetic and non-diabetic patients with asymptomatic coronary artery disease: a cross-sectional study
Source: Cardiovasc Diabetol. 2017 Aug 8;16:96. doi: 10.1186/s12933-017-0577-8 (PMC5549371; doi:10.1186/s12933-017-0577-8)

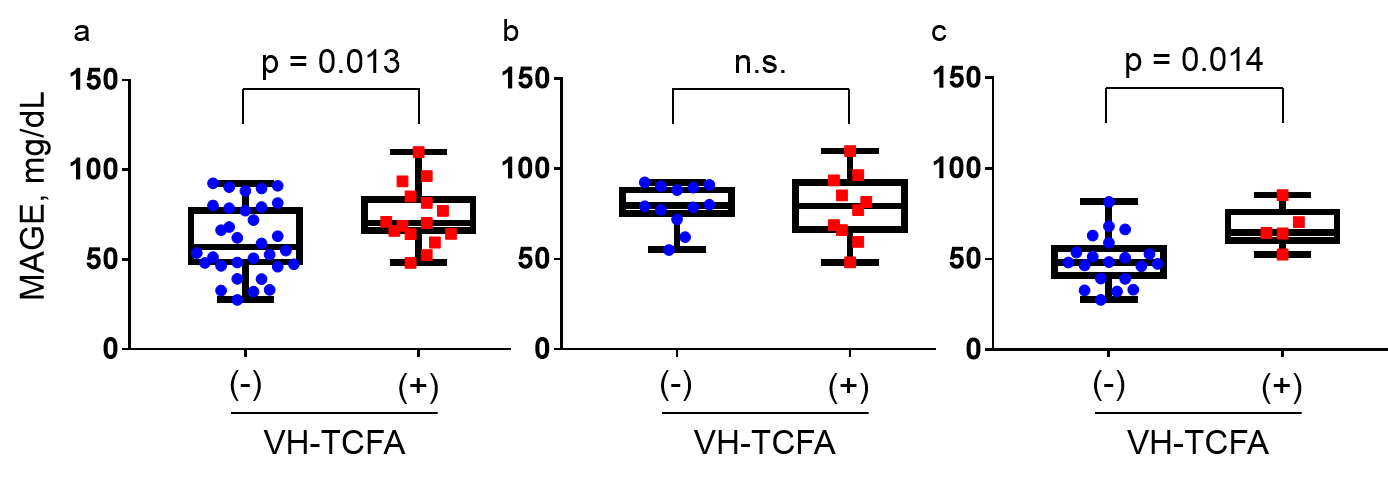

Supplement: Supplementary file 1 — Additional file 1. Relationship between MAGE and VH-TCFA prevalence. Boxplots show MAGE in all patients (a), DM patients (b), and non-DM patients (c) in the presence or absence of VH-TCFA. Error bars represent the minimum to maximum values. DM diabetes mellitus, MAGE mean amplitude of glycemic excursion, VH-TCFA virtual histology thin-cap fibroatheroma. [file 12933_2017_577_MOESM1_ESM.tif]
